# B cell-mediated antigen presentation promotes adverse cardiac remodeling in chronic heart failure

**DOI:** 10.21203/rs.3.rs-4536350/v1

**Published:** 2024-06-24

**Authors:** Jana P. Lovell, Carolina Duque, Sylvie Rousseau, Aashik Bhalodia, Kevin Bermea, Charles D. Cohen, Luigi Adamo

**Affiliations:** 1 Division of Cardiology, Department of Medicine, Johns Hopkins University School of Medicine, Baltimore, Maryland.

## Abstract

Cardiovascular disease remains the leading cause of death worldwide. A primary driver of cardiovascular mortality is ischemic heart failure, a form of cardiac dysfunction that can develop in patients who survive myocardial infarction. Acute cardiac damage triggers robust changes in the spleen with rapid migration of immune cells from the spleen to the heart. Activating this “cardio-splenic” axis contributes to progressive cardiac dysfunction. The cardio-splenic axis has, therefore, been identified as a promising therapeutic target to prevent or treat heart failure. However, our understanding of the precise mechanisms by which specific immune cells contribute to adverse cardiac remodeling within the cardio-splenic axis remains limited. Here, we show that splenic B cells contribute to the development of heart failure via MHC II-mediated antigen presentation. We found that the adoptive transfer of splenic B cells from mice with ischemic heart failure promoted adverse cardiac remodeling and splenic inflammatory changes in naïve recipient mice. Based on single-cell RNA sequencing analysis of splenic B cells from mice with ischemic heart failure, we hypothesized that B cells contributed to adverse cardiac remodeling through antigen presentation by MHC II molecules. This mechanism was confirmed using transgenic mice with B cell-specific MHC II deletion, and by analyzing circulating B cells from humans who experienced myocardial infarction. Our results broaden our understanding of B lymphocyte biology, reshape current models of immune activation in response to myocardial injury, and point towards MHC II-mediated signaling in B cells as a novel and specific therapeutic target in chronic heart failure.

## Introduction:

Acute myocardial infarction (MI) triggers a robust activation of inflammatory responses (1,2). These inflammatory responses are physiologic and necessary to limit tissue injury and induce tissue repair (3,4). However, sustained inflammation contributes to adverse cardiac remodeling and thus, to the development and progression of heart failure (5). Despite advancements in our understanding of the relationship between chronic inflammation and heart failure, clinical trials investigating immunomodulatory therapies in heart failure have yielded largely unsuccessful results. The failure of these therapies is due partly to the inability to selectively target maladaptive versus protective inflammatory responses post-MI (6).

As such, improved understanding of the sustained maladaptive inflammatory responses contributing to the pathogenesis of chronic heart failure is needed. Prior murine and human data indicate that acute MI induces splenic remodeling and mobilization of immune cells from the spleen to the injured myocardium (7). Activation of this “cardio-splenic axis” has been shown to contribute to adverse cardiac remodeling and the development of chronic heart failure (8). Therefore, the cardio-splenic axis has been identified as a promising therapeutic target for developing immunomodulatory treatments for heart failure. However, our mechanistic understanding of the processes by which specific splenic immune cells contribute to adverse cardiac remodeling remains limited.

Within the spleen of most vertebrates, B cells comprise the majority of immune cells. B cells are also prevalent within the heart at baseline and can recirculate between the spleen and the heart (8–10). Growing evidence shows that B cells are involved intricately with cardiac function and dysfunction, and following acute MI, contribute to the development of ischemic heart failure (11–15). However, to date, the potential role of B cells within the cardio-splenic axis of heart failure has not been systematically investigated.

In this study, we address this knowledge gap. We present evidence indicating that B cells play an essential role within the cardio-splenic axis of chronic heart failure. Analyzing the mechanistic basis of this observation, we have found that B cell-mediated antigen presentation drives adverse cardiac remodeling after MI.

## Results

### Splenic B cells contribute to adverse cardiac remodeling after ischemic myocardial injury.

**Figure 1.**
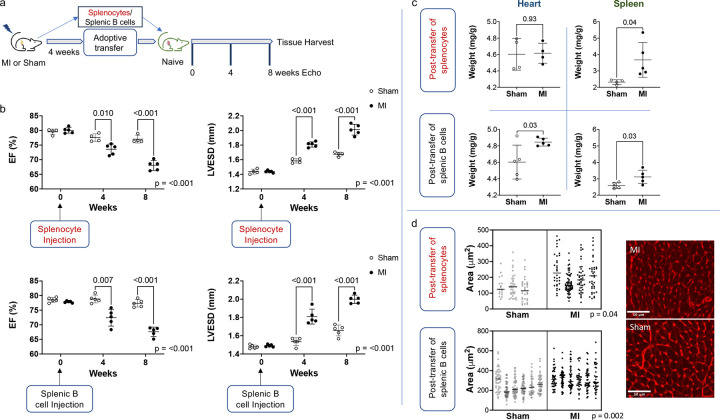
Splenic B cells promote adverse cardiac remodeling after ischemic myocardial injury. a) Schema for adoptive transfer studies. Four weeks following permanent coronary artery ligation or sham surgery, unfractionated splenocytes or isolated splenic B cells were transferred to naïve recipient mice. Recipient mice underwent serial transthoracic echocardiography over 8 weeks followed by tissue harvest. b) On echocardiography, adoptive transfer of splenocytes or isolated splenic B cells from heart failure (HF) mice resulted in a reduction in left ventricular ejection fraction (LVEF) and an increase in left ventricular end-systolic diameter (LVESD) in naïve recipient mice over eight weeks. c) Gravimetric data revealed significantly larger hearts and spleens (normalized to body weight) in naive mice eight weeks post-adoptive transfer of isolated splenic B cells from HF mice relative to recipients of B cells from sham-operated mice. d) Representative images of WGA staining of hearts from recipients of isolated splenic B cells from HF or sham-operated mice. Cardiomyocyte area was increased in recipients of splenocytes or isolated splenic B cells from HF mice relative to sham recipients. n = 5–6 male mice per group. Mean values ± SD are represented.

To investigate the role of splenic B cells in the adverse cardiac remodeling of ischemic heart failure (HF), we designed adoptive transfer experiments. We induced ischemic HF in mice via permanent ligation of the left anterior descending coronary artery. Four weeks after MI, unfractionated splenocytes or purified splenic B cells were transferred into naïve mice via intravenous injection. Splenic immune cells isolated from sham-operated animals were used as controls. The naïve recipient mice were monitored for eight weeks after adoptive transfer (Figure 1A).

We first investigated the effects of adoptive transfer of unfractionated splenocytes. Consistent with previously published data by Ismahil et al. (16), we found the adoptive transfer of splenocytes from mice with ischemic HF resulted in adverse cardiac remodeling in recipient mice. On serial echocardiography, recipients of HF splenocytes had reduced ventricular ejection fraction (LVEF) and increased ventricular end-systolic diameter (LVESD) eight weeks post adoptive transfer compared to recipients of splenocytes from sham-operated mice (Figure 1B; mean LVEF 68.1±1.7% vs 77.0±1.3% HF vs. sham recipient, q<0.001 at 8 weeks; mean LVESD 2.01±0.7mm vs. 1.67±0.02mm HF vs. sham recipient, q<0.001at 8 weeks).

To investigate the role of splenic B cells within the cardio-splenic axis following ischemic myocardial injury, we then performed adoptive transfer of purified splenic B cells from HF or sham-operated mice (≈95% pure, Supplementary Figure 1). Over the eight-week period, the adoptive transfer of isolated HF splenic B cells produced a degree of adverse remodeling similar to that observed with the adoptive transfer of unfractionated splenocytes (Figure 1B, bottom panels). Recipients of splenic B cells from mice with ischemic HF experienced a progressive reduction in LVEF and progressive LV dilatation (Figure 1B; mean LVEF 67.6±1.6% vs. 77.3±1.3% HF vs. sham recipient, q<0.001 at 8 weeks; mean LVESD 2.00±0.05mm vs. 1.66±0.06mm HF vs. sham recipient, q<0.001 at 8 weeks).

Adverse cardiac remodeling in naïve recipients of splenocytes and splenic B cells isolated from HF mice was indicated further by gravimetric analyses of the heart and spleen and wheat-germ agglutinin (WGA) staining of myocardial sections. Gravimetric analysis revealed increased splenic and cardiac weights in recipients of HF splenic B cells compared to recipients of cells from sham-operated mice eight weeks following adoptive transfer (Figure 1C; for purified splenic B cells, spleen mean 3.11±0.4 vs 2.59±0.2 mg/g, p = 0.03; heart mean 4.85±0.05 vs 4.60±0.2 mg/g, p = 0.03; Extended Data Figure 1 reports combined data from multiple independent experiments). WGA-based analysis of myocardial cross-sections showed that the gravimetric changes in the heart were associated with a significant degree of cardiomyocyte hypertrophy (Figure 1D; p = 0.002).

### Adoptively transferred splenic B cells are distributed between the spleen and heart and promote changes in splenic immune cells post-MI.

**Figure 2.**
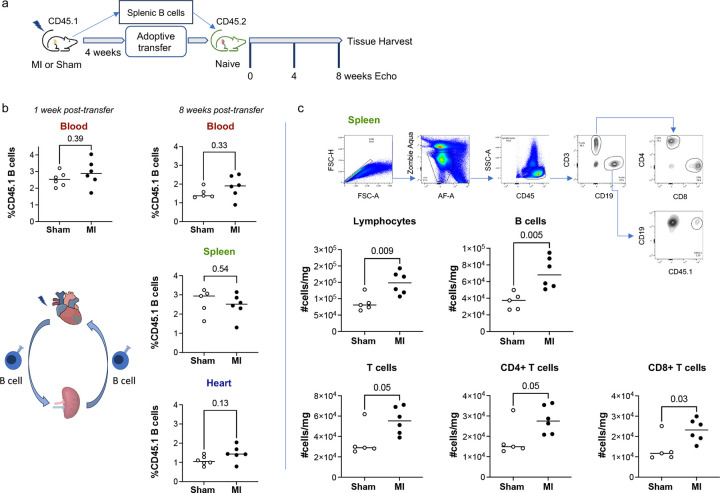
Adoptively transferred splenic B cells migrate to the heart and spleen of recipients, and post-MI, induce inflammatory changes in the spleen of naïve mice. a) Schema for adoptive transfer of isolated splenic CD45.1 B cells 4 weeks following LAD ligation or sham surgery to naïve CD45.2 mice. b) Flow cytometry of peripheral blood from naïve recipient mice revealed the presence of donor CD45.1 B cells 1 week and 8 weeks following adoptive transfer. Donor CD45.1 B cells were also present in the spleen and heart of CD45.2 mice 8 weeks after adoptive transfer. c) Representative gating strategy for flow cytometry of spleens from recipient mice 8 weeks post-adoptive transfer of splenic B cells. Adoptive transfer of splenic B cells from HF mice resulted in increases in lymphocytes, B cells, and CD8+ T cells relative to recipients of sham splenic B cells. The number of cells per mg spleen is reported. n = 6 male mice per group. Median values are represented by horizontal lines.

To investigate the dynamics of adoptively transferred splenic B cells, we repeated the adoptive transfer experiments using animals carrying the CD45.1 isoform as donors and animals carrying the CD45.2 isoform as recipients (Figure 2A). Donor CD45.1 B cells were present in the peripheral blood of naïve recipient mice at one week and eight weeks following adoptive transfer (Figure 2B; ~3% and ~2% of circulating B cells donor-derived at one and eight weeks, respectively). Flow cytometry of the spleen and heart eight weeks post-adoptive transfer also revealed the presence of donor CD45.1 B cells, indicating the re-distribution of donor B cells from both sham-operated and HF mice to the spleens and hearts of recipients (Figure 2B; Extended Data Figure 2).

We used multicolor flow cytometry to assess the effects of splenic B cells isolated from HF mice on the splenic and myocardial immune populations of recipient mice. At the splenic level, adoptive transfer of splenic B cells from mice with chronic ischemic HF was associated with increases in the numbers of total splenic lymphocytes, B cells, and CD8 T cells and with a trend towards an increase in total splenic CD3 T cells and CD4 T cells relative to recipients of splenic B cells from sham-operated mice (Figure 2C). We did not detect differences in cardiac immune cell populations between recipients of splenic B cells from mice with ischemic HF and recipients of splenic B cells from sham-operated mice (Extended Data Figure 3).

### Ischemic myocardial injury results in dysregulation of antigen processing and presentation pathways

**Figure 3.**
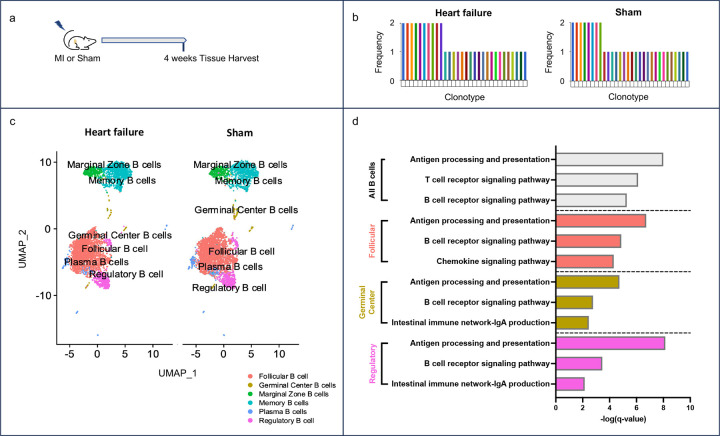
Acute myocardial injury activates antigen processing and presentation pathways in splenic B cells. a) Schema for scRNA sequencing of B cells from mice 4 weeks following permanent coronary ligation or sham surgery. b) V(D)J analysis of splenic B cells. Myocardial infarction (MI) did not induce splenic B cell clonal expansion. c) UMAP plots of splenic B cells 4 weeks post-MI (“heart failure”) or sham surgery. Splenic B cells were classified into B cell sub-types. d) KEGG pathway analysis of differentially expressed genes in splenic B cells (p<0.05) revealed significant dysregulation of antigen processing and presentation in multiple B cell subtypes post-MI. Top 3 dysregulated immune system pathways are reported for specified B cell subtypes. n = 4–5 mice per group.

To explore the mechanistic relationship between the adoptive transfer of splenic B cells from mice with ischemic HF and adverse cardiac remodeling in naïve mice, we performed single-cell RNA sequencing (scRNAseq) on B cells isolated from mice with ischemic HF (four weeks following post-MI) or controls (mice post-sham surgery; Figure 3A). We first focused on splenic B cells. V(D)J analysis did not reveal an expansion of specific clonotypes in mice with ischemic HF, suggesting that MI did not trigger splenic B cells clonal expansion (Figure 3B). UMAP plots were then used to visualize the clustering of gene expression profiles of splenic B cells (Figure 3C). Comparative gene expression analysis using p<0.05 revealed a list of 334 genes differentially expressed in splenic B cells from HF mice. To evaluate the biological relevance of these differentially expressed genes (DEGs), we performed pathway analyses using KEGG pathway annotation (17,18). Antigen processing and presentation was the top biological pathway identified as dysregulated in chronic HF (q-value = 1.2E-8; Figure 3D). Within the spleen, B cells can be classified as follicular, germinal center, marginal zone, memory, regulatory, and plasma B cells based on specific cell markers (19,20). Further evaluation of B cell subtypes revealed significant dysregulation of the antigen processing and presentation pathway in the follicular, germinal center, regulatory, plasma, and memory B cells (Figure 3D) but not in marginal zone B cells (Extended Data Table 1).

To further corroborate our findings, we expanded our analysis to myocardial B cells and peripheral blood B cells in animals with both recent myocardial injury (four days post-MI) and chronic heart failure (four weeks post-MI). These analyses also pointed towards antigen processing and presentation as one of the top dysregulated pathways in myocardial and circulating B cells in response to myocardial injury. More specifically, antigen processing and presentation was dysregulated in multiple cardiac and peripheral blood B cell subtypes from mice with chronic heart failure (Extended Data Figures 4 and 5), with an especially strong signal in myocardial and circulating follicular B cells (Extended Data Figures 4C and 5C). Recent MI was also associated with significant dysregulation of antigen processing and presentation in splenic, cardiac, and peripheral blood B cells with a predilection for specific B cell subtypes (Extended Data Figures 6–8). Lists of the DEGs associated with antigen processing and presentation in heart failure mice and mice with recent MI are provided within the spreadsheets included in Supplementary Table 1.

### B cells promote adverse cardiac remodeling in ischemic heart failure via MHC class II-mediated antigen presentation.

**Figure 4.**
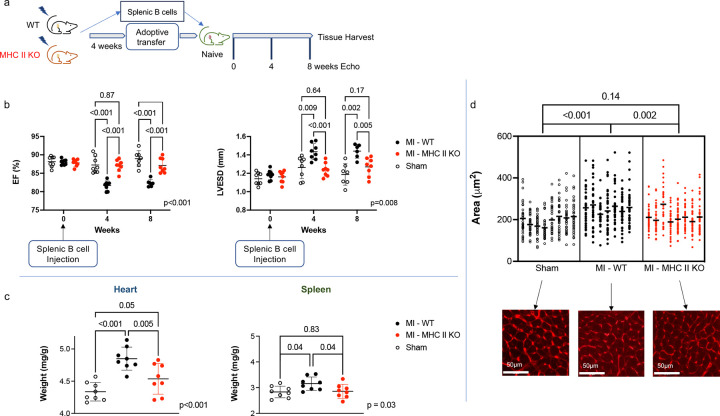
Isolated splenic B cells deficient in MHC class II do not transfer adverse cardiac remodeling to recipient mice. a) Schema for adoptive transfer studies of isolated splenic B cells from *Cd19*^*tm1(cre)Cgn/−*^*H2-Ab1*^*b*-tm1Koni/ *b*-tm1Koni^ mice and wildtype mice following permanent coronary artery ligation or sham surgery. b) Naïve recipients of splenic B cells with MHC II deletion from post-MI mice did not experience adverse cardiac remodeling on serial echocardiography over an 8-week period post-adoptive transfer. c) Gravimetric data of recipient mice normalized to body weight. Recipient mice of B cells deficient in MHC II from post-MI mice did not experience splenocyte or cardiac remodeling by gravimetric analysis. d) Representative images of WGA staining of hearts from recipient mice. Transfer of isolated MHC II-deficient splenic B cells did not induce cardiomyocyte hypertrophy. n = 8 male mice per group. Mean values ± SD are represented.

Collectively, our transcriptomic data suggested strongly that splenic B cells might modulate adverse cardiac remodeling after MI via major histocompatibility complex class II (MHC II) mediated antigen presentation. B cells have three main functions: antibody production, cytokine secretion, and antigen presentation. It has been reported that B cells may contribute to adverse cardiac remodeling following ischemic MI via the production of autoantibodies (5,11) or chemokine-mediated mobilization of monocytes from the spleen to the heart (21). However, to our knowledge, B cell-mediated antigen presentation has never been implicated in the pathogenesis of cardiac dysfunction. To investigate this possible mechanism, we developed a transgenic mouse model with B cell-specific MHC II deletion (*Cd19*^*tm1(cre)Cgn/−*^*H2-Ab1*^*b*-tm1Koni/*b*-tm1Koni^) (22) and repeated our adoptive transfer experiments (Figure 4A).

**Figure 5.**
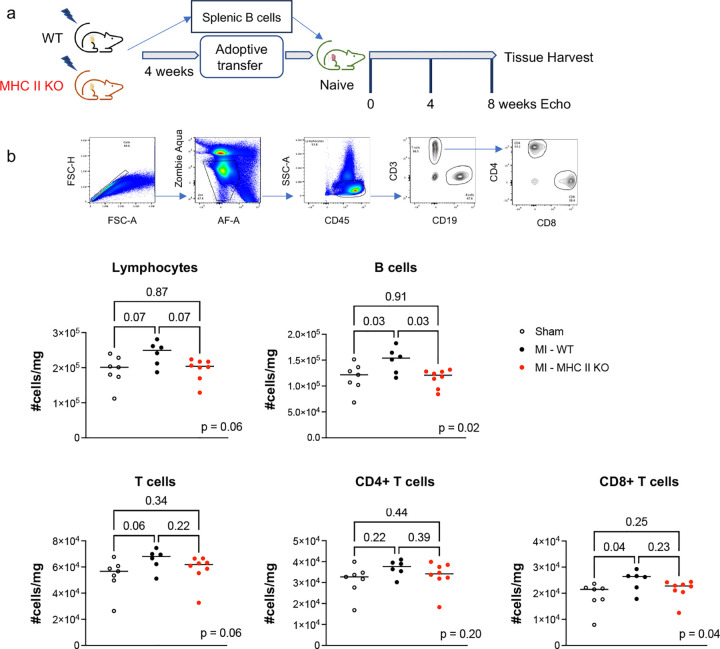
Isolated splenic B cells deficient in MHC class II do not induce inflammatory changes in the spleen of naïve mice post-adoptive transfer. a) Schema for adoptive transfer studies of isolated splenic B cells from *Cd19*^*tm1(cre)Cgn/−*^*H2-Ab1*^*b*-tm1Koni/ *b*-tm1Koni^ mice and wildtype mice following permanent coronary artery ligation or sham surgery. b) Representative gating strategy for flow cytometry of spleens from recipient mice 8 weeks post-adoptive transfer of splenic B cells. Adoptive transfer of splenic B cells with MHC II deletion from post-MI mice did not induce changes in immune cell populations in the spleens of recipient mice. n = 8 male mice per group. Median values are represented by horizontal lines.

Four weeks following MI, we performed adoptive transfer of MHC II-deficient splenic B cells into naïve wild-type (WT) mice. Flow cytometry analysis confirmed the persistent absence of MHC II on B cells at the time of adoptive transfer (Extended Data Figure 9). Adoptive transfer of splenic B cells from WT HF mice or sham-operated mice served as positive and negative controls, respectively. Splenic B cells with MHC II deletion isolated from post-MI mice did not induce adverse cardiac remodeling upon adoptive transfer (Figure 4B; mean LVEF 87.10±1.9% vs. 88.96±2.2% MHC II-deficient post-MI vs. WT sham recipient, q = 0.11 at 8 weeks; mean LVESD 1.27±0.1mm vs. 1.19±0.1mm MHC II-deficient post-MI vs. WT sham recipient, q = 0.17 at 8 weeks). Gravimetric data revealed borderline increases in cardiac weights in the absence of splenic hypertrophy following the transfer of MHC II-deficient post-MI splenic B cells (Figure 4C; spleen mean 2.86±0.3 vs. 2.83±0.2 mg/g MHC II-deficient post-MI vs. WT sham recipient, q = 0.83; heart mean 4.54±0.2 vs. 4.34±0.1 mg/g MHC II-deficient post-MI vs. WT sham recipient, q = 0.05). Cardiomyocyte hypertrophy was also markedly reduced in recipients of MHC II-deficient post-MI splenic B cells compared to recipients of WT HF splenic B cells (Figure 4D). Immune cell populations in the spleens of recipient mice were then evaluated via flow cytometry. The recipients of isolated WT HF splenic B cells showed similar increases in inflammatory cell populations as seen in our prior experiments. (Figure 5) However, the splenic immune cell populations of recipients of MHC II-deficient post-MI splenic B cells were comparable to those observed in recipients of sham splenic B cells, indicating reduced splenic inflammatory changes in response to MHC II-deficient post-MI splenic B cells (Figure 5).

### Ischemic heart failure in humans is associated with dysregulation of antigen processing and presentation pathways in B cells.

To evaluate the clinical relevance of our observations, we next sought to assess dysregulated biological pathways in B cells isolated from patients who experienced ischemic myocardial injury. Since our murine data showed that changes in splenic B cells were reflected in peripheral blood B cells and isolation of splenic B cells from patients was not feasible, we performed a focused analysis of publicly available peripheral blood scRNAseq data from patients with ST-elevation myocardial infarction (STEMI) (23) and patients with established ischemic HF relative to healthy patients (24). In both datasets, we identified peripheral blood B cells and performed differential gene expression analysis.

**Figure 6.**
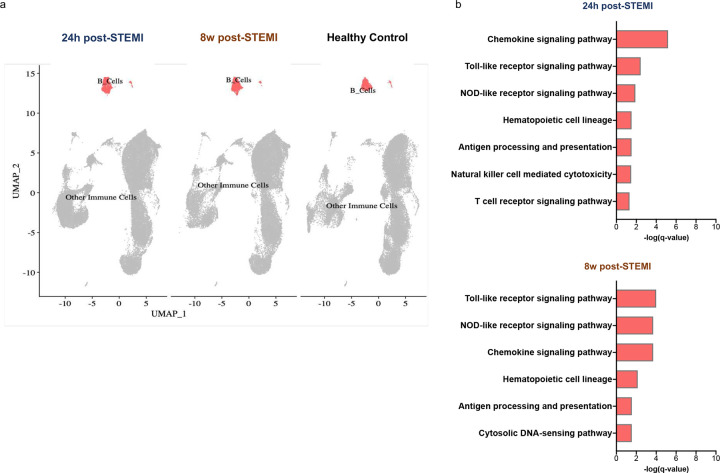
Antigen processing and presentation is dysregulated in circulating B cells in human patients post-STEMI. a) scRNA sequencing of peripheral blood B cells from 38 human patients 8 weeks post-STEMI and 38 healthy controls. UMAP plots are shown with B cells identified. b) KEGG pathway analysis of differentially expressed genes (log2fold-change ≥1, p<0.05) revealed significant dysregulation of antigen processing and presentation in peripheral B cells of patients 24 hours and 8 weeks post-STEMI. All immune system pathways with q-value <0.05 are listed. NOD= nucleotide oligomerization domain.

**Figure 7.**
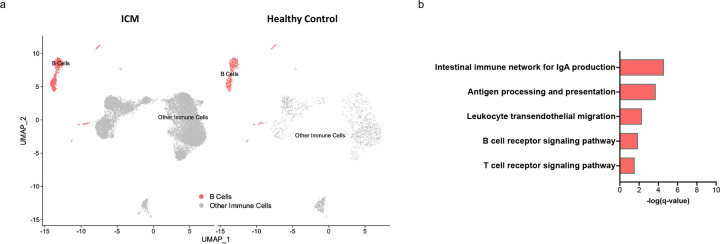
Antigen processing and presentation is dysregulated in circulating B cells in humans with ischemic cardiomyopathy. a) scRNA sequencing of peripheral blood B cells from 3 human patients with ischemic cardiomyopathy (ICM) and 1 healthy patient. UMAP plots are shown with B cells identified. b) KEGG pathway analysis of differentially expressed genes (log2fold-change ≥0.58 p<0.05) revealed significant dysregulation of antigen processing and presentation in peripheral B cells from ICM patients. All immune system pathways with q-value <0.05 are listed.

The STEMI dataset included peripheral blood scRNAseq data of 38 patients 24 hours and eight weeks post-STEMI in addition to 38 healthy controls (23). Focusing on B cells, we performed DEG analysis comparing post-STEMI patients at both timepoints with healthy controls. Using a log2fold change threshold 31 and p<0.05, we identified 459 DEGs at 24 hours post-MI and 493 DEGs eight weeks post-MI. KEGG pathway analysis of these DEGs highlighted antigen processing and presentation as a dysregulated pathway both at 24 hours post-MI (q = 0.03) and eight weeks post-M (q = 0.03), (Figure 6). In the second dataset of three patients with established ischemic heart failure and one matched healthy control (24), we identified 305 DEGs using a log2fold change threshold 30.58 and p<0.05. We used a more conservative fold change threshold given the smaller size of this dataset. Antigen processing and presentation was identified again as a top dysregulated pathway in patients with ischemic HF (q = 1.85E-4), (Figure 7). Complete lists of the DEGs within the antigen processing and presentation pathway are provided in Supplementary Table 2.

## Discussion

In this study, we report for the first time that MHC II-dependent B cell-mediated antigen presentation contributes to adverse cardiac remodeling after ischemic myocardial injury. This finding broadens our understanding of B cell biology and points to B cell-mediated antigen presentation as a novel and specific therapeutic target to prevent and treat heart failure.

Because of the abundant data supporting its clinical relevance, we focused primarily on the cardio-splenic axis of ischemic heart failure in this study. Swirski et al. were the first to describe rapid mobilization of monocytes from the spleen to the heart in murine models of ischemic MI (7). Ismahil et al. then found that splenectomy post-MI reduces adverse cardiac remodeling, while the adoptive transfer of splenocytes from mice with ischemic heart failure is sufficient to produce adverse cardiac remodeling in naïve mice (16). Consistent with these findings, autopsies of patients with fatal MI revealed an accumulation of monocytes at the infarcted myocardium with concurrent monocyte depletion in the spleen (25,26). In another study of 22 patients with ischemic MI, fludeoxyglucose-18 (FDG) positron emission tomography (PET) revealed increased splenic inflammation post-MI relative to control patients (27). Increased FDG uptake in the spleen was associated further with risk of future adverse cardiac events in a larger cohort of patients undergoing FDG-PET (27).

While B cells are the most prevalent splenic immune cell across multiple vertebrates, there is limited data on the role of B cells within the cardio-splenic axis. To address this knowledge gap, we used the adoptive transfer model established by Ismahil et al., which allows uniquely for the isolation of the functional effects of specific immune cell types within the cardio-splenic axis of ischemic heart failure (16). We found that the adoptive transfer of splenic B cells from mice with ischemic heart failure results in adverse cardiac remodeling with evidence of LV dilatation, reduced LV systolic function, and cardiomyocyte hypertrophy in naïve mice. Our findings thus provide definitive evidence that B cells play a significant role within the cardio-splenic axis of ischemic heart failure. Since the adoptive transfer of purified B cells recapitulated the effect of the adoptive transfer of unfractionated splenocytes, our findings suggest that B cells might be critical players within the cardio-splenic axis.

We next found that adoptively transferred splenic B cells re-distributed between the heart, spleen, and peripheral blood of recipient mice, suggesting recirculation of transferred splenic B between the heart and spleen, consistent with B cell transit dynamics at baseline (8). We then observed that MI triggered the dysregulation of the antigen processing and presentation signaling pathway in splenic B cells four weeks following injury, indicating that MI induces long-lasting changes in B cell function. The dysregulation of antigen processing and presentation was observed across splenic, myocardial, and circulating B cells, corroborating further the idea that B cells recirculate between the spleen and the heart through the peripheral blood after MI. However, we did not observe substantial B cell clonal expansion post-MI, suggesting that B cells likely acquire and present antigens via B cell receptor (BCR)-independent mechanisms rather than through engagement of the BCR (28). Since B cells can present antigens to other immune cells via MHC II molecules independent of BCR antigen binding (28), we developed a transgenic mouse model with B cell-specific deletion to MHC II to investigate B cell-mediated antigen presentation within the cardio-splenic axis.

The adoptive transfer of post-MI B cells with either intact or deficient MHC II expression highlighted that B cells contribute primarily to adverse cardiac remodeling along the cardiosplenic axis via MHC II-mediated antigen presentation. MHC II deletion abrogated evidence of adverse cardiac remodeling on serial echocardiography of recipient mice. There were, however, some indications of mild effects of post-MI MHC II-deficient splenic B cells within recipient mice, with trends observed towards increased cardiac weight and cardiomyocyte area. This mild phenotype may be due to residual low-level expression of MHC II on B cells in our transgenic knockout model or indicate additional mechanisms by which B cells contribute to adverse cardiac remodeling post-MI (29).

Prior studies have shown that B cells can modulate adverse cardiac remodeling after MI through various mechanisms. Following MI, splenic B cells upregulate the chemokine CCL7, which promotes monocyte mobilization from the spleen to the injured myocardium, contributing to adverse cardiac remodeling (11,21). Disruption in Notch signaling in B cells, which is associated with splenic marginal zone B (MZB) cell deficiency, reduces adverse cardiac remodeling after MI (21,30). B cells can also ameliorate maladaptive inflammation post-MI via secretion of the anti-inflammatory cytokine interleukin-10 or production of acetylcholine (12,13). There is additional evidence suggesting that B cell production of autoantibodies could contribute to the pathogenesis of chronic heart failure, given the presence of circulating anti-cardiac antibodies in heart failure patients (5,31,32). Our study complements this literature, indicating that B cell-mediated antigen presentation plays an important role in heart failure.

Our scRNAseq analyses of peripheral blood B cells from patients after ischemic myocardial injury showed dysregulation of antigen processing and presentation in both the acute and chronic phases of ischemic MI, strongly suggesting that our findings are relevant to human biology. Several of the genes differentially expressed within this pathway were shared between mice and humans, including multiple MHC complexes, CD74, which stabilizes MHC II molecules (33), and HSPs, which are critical modulators of effector response after antigen presentation (34,35). B cells present antigens via MHC II molecules to CD4 T cells and, under specific conditions CD8 T cells (36,37), resulting in T cell activation. Prior murine data have implicated CD4 T cells and CD8 T cells in adverse cardiac remodeling following myocardial injury (38–41). Within the context of the cardio-splenic axis, the adoptive transfer of splenic CD4 T cells from mice with ischemic HF is sufficient to induce adverse LV remodeling in naïve recipients (38). In this present study, we found that the adoptive transfer of post-MI B cells with intact MHC II expression induced an expansion of splenic T cells in naïve mice. At the same time, these changes were absent in recipients of B cells with MHC II deletion. B cells have been implicated as the dominant antigen-presenting cell activating CD4 T cells in non-cardiovascular disease models (42,43). We thus hypothesize that antigen presentation by B cells is an obligate requirement for generating cardiotoxic T cells after ischemic myocardial injury.

In summary, our findings indicate that B cells recirculating between the heart and spleen drive adverse cardiac remodeling of the injured heart via MHC II-mediated antigen presentation. This observation challenges current models of the interaction between immune cells and the heart after myocardial injury and expands current understanding of B cell biology (2,44–46). While further studies will be needed to fully elucidate the specific B cell subtype driving adverse cardiac remodeling and the functional effect of B cell-mediated antigen presentation within the immune response elicited by myocardial damage, our findings identify MHC II-dependent B cell-mediated antigen presentation as a novel and specific therapeutic target to prevent and treat heart failure.

## Methods:

All studies were approved by the Institutional Animal Care and Use Committee (ACUC) of the Johns Hopkins University School of Medicine, under protocol number M023M238.

### Mouse Models and Surgical Protocol:

Male 10–14 weeks-old mice were used for all experiments unless otherwise specified. All mice were purchased from Jackson Laboratory (Bar Harbor, ME). The following strains were used: C57BL/6J strain N. 000664, B6.SJL-*Ptprc*^*a*^ *Pepc*^*b*^/BoyJ (CD45.1) strain N. 002014, B6.129P2(C)-*Cd19*^*tm1(cre)Cgn*^/J strain N. 006785, and B6.129X1-*H2-Ab1*^*b-tm1Koni*^/J strain N. 013181. To generate mice with B cell-specific MHC class II deletion, B6.129P2(C )-*Cd19tm1(cre)Cgn*/J and B6.129X1-*H2-Ab1b-tm1Koni*/J mice were bred for target *Cd19tm1(cre)Cgn/-H2-Ab1*^*b*-tm1Koni/ *b*-tm1Koni^ mice. Mice underwent left thoracotomy with either permanent ligation of the left anterior descending (LAD) coronary artery or sham intervention.

### Adoptive Transfer Studies:

Mice were euthanized four weeks following left thoracotomy with either permanent ligation of the LAD coronary artery or sham surgery. Spleens were harvested and placed into a 35mm tissue culture dish with phosphate-buffered saline (PBS). Splenocytes were isolated as previously published by Ismahil et al.(16). The spleen was minced finely using scissors and the end of a syringe plunger. The solution was then pipetted through a 40μm cell strainer into a sterile 50mL tube. The tube was centrifuged at 250g for 5 minutes at 4°C. After decanting the supernatant, the pellet was resuspended with 5mL of ACK lysis buffer (Quality Biological) for 5 minutes at room temperature. After 5 minutes of lysis, 30mL of PBS was added to each tube and each tube was centrifuged for 5 minutes. The splenocytes were then resuspended in sterile 0.9% sodium chloride for a final concentration of ~1×10^8^ total splenocytes in 150μL per retro-orbital injection into naïve recipient mice.

For adoptive transfer studies with purified B cells, splenic B cells were isolated according to the MojoSort^™^ Mouse Pan B Cell Isolation Kit (BioLegend) protocol, with slight modifications. Briefly, after mincing and filtering each spleen as described above, the pellet of each spleen was resuspended with 4mL of Mojo buffer and then filtered again into FACS tubes. Each tube containing one spleen was then centrifuged at 250g for 5 minutes at 4°C. After decanting the supernatant, the pellet was resuspended with 2mL Mojo buffer. 200μL of primary antibody cocktail was then added to each tube and incubated for 15 minutes, followed by incubation with 200μL of Nanobeads for an additional 15 minutes. After adding 1.5mL of Mojo buffer, the tube was placed into a MojoSort magnet for 5 minutes. The cell suspension was decanted into a fresh FACS tube. This step was repeated to increase the yield of isolated B cells. The suspension of each spleen was then centrifuged and each pellet was resuspended in FACS buffer. The splenic B cell solutions from mice of similar conditions were combined into a single tube and then centrifuged prior to final resuspension in sterile 0.9% sodium chloride for retro-orbital injection of ~1×10^7^ B cells in 150μL into naïve recipient mice. A small sample was obtained pre- and post-antibody incubation to evaluate purity and yield by flow cytometry (Supplementary Figure 1). Purified splenic B cells consistently had >95% purity.

### Echocardiography:

Mouse transthoracic echocardiography was performed in conscious mice prior to retro-orbital injection to obtain a baseline assessment of cardiac structure and function, and then 4- and 8 weeks post-injection. Echocardiography was performed using a VisualSonics Vevo 2100 Imaging System. M-mode parasternal short images were obtained. Left-ventricular ejection fraction (LVEF) and left ventricular end-systolic diameter (LVESD) were measured using Vevo Lab software v5.8.2.

### Gravimetric and Histological Analyses:

Eight weeks after the adoptive transfer, recipient mice were euthanized, and hearts and spleens were dissected and weighed. Tibia length and body weight were recorded to normalize the weights of each mouse heart and spleen. Heart tissue was fixed with 10% neutral buffered formalin, paraffin-embedded, and stained with WGA using standard protocols.

### Isolation of Myocardial Mononuclear Cells:

To analyze myocardial immune cells via flow cytometry, the heart was digested as previously described with slight modifications (47). Each heart was perfused with 3mL of Hank’s Buffered Salt Solution (HBSS) with calcium and magnesium infused into the left ventricle over 1 minute with aortic clamping. Hearts were then dissected and cut transversely approximately at the mid-papillary muscle level, or approximately ~5mm below the atrioventricular groove. The superior section was used for flow cytometry and the apical section was used for histological analysis. The heart tissue for flow cytometry was finely minced and 60mg was suspended in 3mL HBSS. Heart tissue was digested with 300U DNAse, 625U collagenase II, and 50U hyaluronidase shaken for 30 minutes at 300rpm and 37°C. At the end of the 30 minutes, additional HBSS was added to each tube to neutralize the digestion. Each tube was then centrifuged at 250g for 5 min at 4°C. The supernatant was decanted, and the pellet was resuspended in 5mL of ACK lysis buffer for 5 minutes at room temperature. The suspension was then diluted with PBS and filtered through a 40μm strainer. Each cell suspension was centrifuged at 250g for 5 min prior to resuspension in FACS buffer and filtering through a 35μm mesh strainer into sterile FACS tubes.

### Isolation of Splenocytes for Flow Cytometry:

The spleens were removed and prepared as described above for adoptive transfer of splenocytes. Following completion of red blood cell (RBC) lysis, cells were resuspended in FACS buffer and filtered through 35μm cell strainers into sterile FACS tubes.

### Isolation of Peripheral Blood Cells for Flow Cytometry:

Peripheral blood (~100μL) was collected via venipuncture of the facial vein into 1.5mL Eppendorf tubes containing 100μL heparin. Red blood cells were lysed with 1mL ACK buffer for 5 minutes at room temperature and then centrifuged for 5 minutes at 250g at 4°C. Cells were resuspended in 500μL FACS buffer then filtered through 35μm cell strainers into FACS tubes.

### Flow Cytometry:

Isolated cell suspensions were stained for 30 minutes on ice with the following fluorescently conjugated antibodies: CD3-FITC (clone 17A2, Invitrogen), CD3-BV605 (17A2, BioLegend), CD19-BV421 (6D5, BioLegend), CD4-Spark NIR 685 (GK1.5, BioLegend), CD8-BV785 (53-6.7, BioLegend), CD45-PerCP/Cyanine5.5 (30-F11, BioLegend), CCR2-APC (QA18A56, BioLegend), CD64-PE/Cy7 (X54–5/7.1, BioLegend), Ly6C-BV650 (HK1.4, BioLegend), MHCII-BV711 (M5/114.15.2, BioLegend), CD11b-Alexa Fluor 700 (M1/70, BioLegend), Ly6G-APC/Cy7 (1A8, BioLegend), CD45.1-PE (A20, BioLegend). All antibodies were added at approximately 1:1000 dilution for heart and spleen flow cytometry and 1:500 for peripheral blood flow cytometry. Dead cells were excluded using the Zombie aqua dye (BioLegend) at 1:500 dilution. Flow cytometry was performed on Cytek Aurora Full Spectrum Flow Cytometric Analyzer. Compensation controls were generated using single-color control samples and UltraComp eBeads (Invitrogen). Gating strategies are shown in individual figures.

### Single Cell RNA (scRNA) Sequencing:

C57BL/6J mice 14–18 weeks-old (n = 16 total) underwent left thoracotomy with either sham surgery (n = 5) or permanent coronary ligation (n = 11). Blood, heart, and spleen were collected 4 weeks post-sham or MI surgery (“heart failure”; n = 6) or 4 days post-MI surgery (“recent MI”; n = 5). Single-cell suspensions for blood, heart, and spleen were prepared as described above. Heart and spleen samples were stained with 2 drops of Vibrant Dye Cycle Violet Ready Flow Reagent (DCV-Invitrogen) for 30 minutes, shaking at 300rpm and 37°C. All samples were stained with propidium iodide (PI), CD45-PE and CD19-APC antibodies for 30 minutes on ice. Hashtag antibodies specific to each condition were also added to cell suspensions at a concentration of 2μL per tube. B cells were sorted using a MoFlo sorter. Equal numbers of the 3 studied conditions (sham, recent MI, heart failure) were mixed to reach a total of 20,000–30,000 B cells that were combined in equal proportions within the same single cell library. Three libraries were prepared: 1) peripheral blood B cells; 2) splenic B cells; and 3) myocardial B cells.

The Chromium Next GEM Single Cell 5’ Kit v2 (10x Genomics) was used to perform single cell transcriptional analyses on B cells in peripheral blood, spleen, and heart. The Chromium Next GEM Single Cell V(D)J Kit was used to assess B cell receptor clonality.

Demultiplexing, barcode processing, single cell 5’ gene counting, and V(D)J transcript sequence assembly and annotation was performed using Cell Ranger (10x Genomics). Gene specific analysis was performed using *Partek^™^ Flow^™^* software, version 11.0. Single cells with total feature counts <200 and >10000 were excluded. Gene expression levels per cell were then normalized to counts per million and log2 transformed. Analyses of differential expressed genes between conditions were performed using DEseq2 with false-discovery rate (FDR) step-up used for multiple test corrections. Lists of genes that exhibited differential expression for the defined threshold between groups were compiled and then analyzed against the Kyoto Encyclopedia of Genes and Genomes (KEGG) database on the Gene Set Enrichment Analysis (GSEA) website (https://www.gsea-msigdb.org; (17,18).

Uniform manifold approximation and projection (UMAP) plots were created on R (v.4.0.2) using Seurat (v.4.1.1) standard parameters unless otherwise noted. Cells were filtered based on their distributions to only include those with 200–3200 unique features for blood, 200–3800 for heart, and 200–3500 for spleen, and < 8% mitochondrial read counts. Counts were then logNormalized and scaled, and the top 2000 most variable genes were identified using the FindVariableFeatures function. Principal component (PC) analysis was performed using RunPCA and the first 15 PCs were used to find the shared nearest neighbors (SNN) using the FindNeighbours function. Clustering was performed using the FindClusters function with a resolution of 0.2 and visualized using a UMAP projection. B cell subtypes were classified using ScType (19) by using a modified list of known murine B cell markers (Supplementary Table 3) and mapping mouse gene symbols to their human counterpart using biomart to be compatible with ScType.

### Human scRNA Sequencing Analysis:

Publicly available scRNA sequencing data of peripheral blood from two cohorts were analyzed: three patients with ischemic cardiomyopathy (ICM) (24) and 38 patients 24 hours and 8 weeks following ST segment elevation MI (STEMI) (48) relative to healthy controls. These data were processed using Seurat as described above with the following modifications: Cells were filtered based on their distributions to only include those with 200–8000 unique features and <5% mitochondrial reads for the ICM dataset, 200 – 4500 unique features and <12% mitochondrial reads for the STEMI v2 dataset, 200 – 3500 unique features and <8% mitochondrial reads for the STEMI v3 dataset. 3000 variable features were used for both human datasets. Both datasets had notable batch effects that were corrected using Harmony. For both datasets, the first 30 PCs were used for the SNN and clustering. A clustering resolution of 0.5 and 0.4 was used for the ICM and STEMI datasets respectively. B cells were identified using known human B cell-specific makers as previously described (Supplementary Table 4) (49). For the larger dataset, B cells were pseudobulked per patient and differential gene expression was performed using DEseq2 controlling for sex and 10x chemistry in the regression model. Subsequent KEGG pathway analyses were performed as described above. Given the size of the smaller dataset, the defined log2fold change threshold used was 30.58 with p<0.05. For the larger dataset, a more stringent log2fold change threshold of 31 was used.

### Statistical Analysis

After assessing normality with Shapiro-Wilk tests, Mann-Whitney or unpaired two-tailed t-tests were used for non-parametric and parametric comparisons, respectively, of two groups. To compare gravimetric data across three or more groups, one-way analysis of variance (ANOVA) was used followed by pairwise comparisons. For flow cytometry analyses of three or more groups, one-way ANOVA or Kruskal-Wallis tests were used for normally distributed or non-Gaussian distributed data, respectively, followed by pairwise comparisons. Serial echocardiographic data were analyzed using two-way ANOVA or mixed-effects analysis with Geisser-Greenhouse correction followed by pairwise comparisons. WGA data were analyzed using nested t-test for two groups or nested one-way ANOVA followed by pairwise multiple comparisons for three groups. All pairwise multiple comparison tests were corrected using the original FDR method of Benjamini and Hochberg. All statistical tests were performed on GraphPad Prism version 9.4.1 for macOS, GraphPad Software, La Jolla California USA, www.graphpad.com (Graph Pad).
